# EXPANDING RURAL ACCESS TO TELE-ENHANCEFITNESS THROUGH COMMUNITY-ENGAGED DESIGN OF A REFERRAL PATHWAY

**DOI:** 10.1093/geroni/igae098.0998

**Published:** 2024-12-31

**Authors:** Elise Hoffman, Elspeth Rensema, Leah Adams, Shannon Jajko, Nancy Gell, Basia Belza, Kushang Patel

**Affiliations:** University of Washington, Boston, Massachusetts, United States; University of Washington, Seattle, Washington, United States; George Mason University, Fairfax, Virginia, United States; University of Washington, Seattle, Washington, United States; University of Vermont, Burlington, Vermont, United States; University of Washington, Seattle, Washington, United States; University of Washington, Seattle, Washington, United States

## Abstract

Rural older adults experience a high burden of chronic pain from osteoarthritis and other chronic conditions. Exercise is a first-line treatment for many painful conditions, but access to evidence-based programs is limited in rural areas. Remotely delivered programs, including Enhance®Fitness (Tele-EF), has high potential to expand access. Therefore, we conducted a series of studies to assess feasibility of delivering Tele-EF to rural older adults with chronic pain. First, we partnered with a rural-serving healthcare system to pilot 12 weeks of Tele-EF in 15 rural older adults with knee osteoarthritis. Median attendance was 91%, 100% of participants were very satisfied with tele-EF, 87% completed the program, and pain and physical functioning improved significantly. Next, we piloted implementation of an exercise prescription and Tele-EF referral pathway for older adults with knee osteoarthritis in a rural-serving primary care clinic. Adoption was low, with 4 patients referred, screened, and enrolled in Tele-EF over 16 weeks. In an effort to improve the referral pathway for a NIH-funded pragmatic trial on rural pain management, we conducted 20 interviews with EF instructors and leaders of rural-serving YMCAs. Preliminary results suggest referral pathways are effective when built into existing clinic and community systems and involve sustained provider outreach. Ongoing community engagement will include focus groups to assess usability of the pathway, prior to its implementation in a pilot of the pragmatic trial. Taken together, current results indicate that delivering EF to rural older adults is feasible, but engaging with rural healthcare systems is critical to reaching this population.

